# Comparative evaluation of ternary amorphous solid dispersions: Identifying optimal excipient systems for enhancing drug solubility

**DOI:** 10.1016/j.ijpx.2025.100461

**Published:** 2025-12-02

**Authors:** Arif Budiman, Lisa Efriani Puluhulawa, Faradila Ratu Cindana Mo’o, Nurain Thomas, Melvern Theodorik S. Biu, Febrina Amelia Saputri, Siti Farah Rahmawati, Diah Lia Aulifa, Salma Amaliah, Agus Rusdin

**Affiliations:** a Department of Pharmaceutics and Pharmaceutical Technology, Faculty of Pharmacy, Universitas Padjadjaran, Sumedang, Indonesia; b Department of Pharmacy, Faculty of Sport and Health, Universitas Negeri Gorontalo, Gorontalo, Indonesia; c Departement of Pharmacy, Faculty of Pharmacy, Universitas Indonesia, Depok, Indonesia; d Department of Pharmacology and Clinical Pharmacy, Institut Teknologi Bandung, Bandung, Indonesia; e Department of Pharmaceutical Analysis and Medicinal Chemistry, Faculty of Pharmacy, Universitas Padjadjaran, Sumedang, Indonesia

**Keywords:** Amorphous solid dispersion, Solubility enhancement, Ternary system, Surfactants, Bioavailability, Molecular interaction

## Abstract

The limited aqueous solubility of numerous active pharmaceutical ingredients (APIs) remains a major barrier to achieving optimal oral bioavailability, therapeutic efficacy, and clinical translation. Amorphous solid dispersion (ASD) systems have emerged as a leading strategy to overcome these biopharmaceutical limitations, with ternary ASDs offering greater formulation flexibility and performance enhancement through the synergistic inclusion of functional third components.

**Aims:**

This review aims to systematically explore and critically analyze the formulation strategies, comparative outcomes, and molecular mechanisms underlying ternary ASDs—specifically Drug:Polymer:Polymer, Drug:Polymer:Surfactant, Drug:Polymer:Excipient, and Drug:Drug:Polymer systems—in improving solubility, dissolution, stability, and pharmacokinetic performance**.** A comprehensive literature search was conducted across Scopus, PubMed, and Web of Science databases for peer-reviewed articles published between 2015 and 2025, focusing on experimental studies evaluating ternary ASDs. Studies were selected based on relevance to solubility enhancement, dissolution profile, in vitro–in vivo correlation, and mechanistic insights at the molecular level. Ternary ASDs demonstrated superior performance over binary systems, particularly those incorporating surfactants, which exhibited the highest solubility enhancement (up to 810.81-fold). Polymer–polymer and polymer–excipient systems also improved dissolution and pharmacokinetic parameters, although with lower magnitude. Mechanistically, ternary ASDs work through micellization, hydrogen bonding, molecular dispersion, and recrystallization inhibition, which collectively maintain supersaturation and improve absorption and bioactivity. Ternary ASD systems represent a scientifically rational and pharmaceutically significant advancement for formulating poorly soluble drugs. Their ability to modulate solubility, dissolution, and pharmacological outcomes through molecular-level interactions underscores their transformative potential in drug delivery. Future research should focus on tailoring ternary components based on physicochemical drug properties and predictive modeling.

## Graphical abstract

**Figure:**
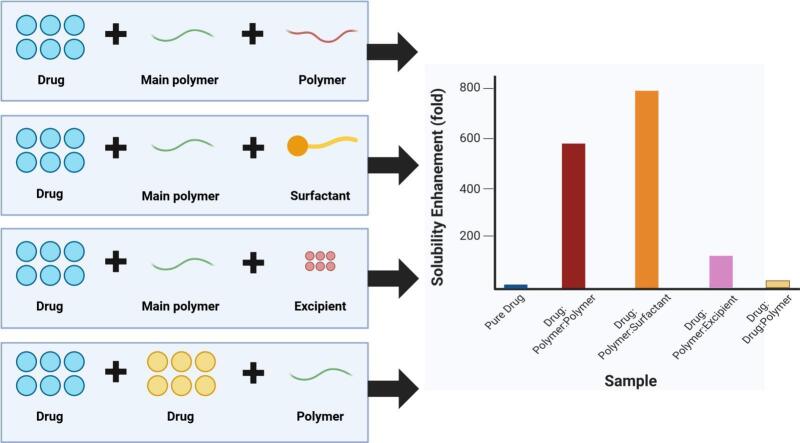

## 1 Introduction

Poor aqueous solubility remains one of the most pervasive challenges in pharmaceutical development, particularly in the formulation of orally administered drugs (Attia and Ghazy, 2023; Bhalani et al., 2022; Salunke et al., 2022). An estimated 40–60 % of new chemical entities (NCEs) identified through high-throughput screening exhibit low water solubility, which directly compromises their dissolution rate, oral absorption, and ultimately, therapeutic efficacy (Budiman et al., 2019; Kawabata et al., 2011; Tran et al., 2019). This solubility-limited bioavailability significantly hinders the clinical translation of promising drug candidates and represents a critical barrier in the drug development pipeline (Di et al., 2012; Jermain et al., 2018; Kumari et al., 2023; Pandi et al., 2020; Shi et al., 2019; Zhuo et al., 2024). Overcoming this limitation requires not only technological innovation but also a deeper mechanistic understanding of formulation strategies that effectively address solubility enhancement at the molecular level (Kosaka et al., 2020; V A, S, et al., 2025).

Currently, several formulation strategies have been developed to address this issue, including salt formation (Gao et al., 2024; Meng et al., 2024; Yarlagadda et al., 2024; Zhang et al., 2024), particle size reduction (Abdollahi et al., 2024; Csicsák et al., 2023; Rashed et al., 2022), lipid-based systems (Alajami et al., 2022; Koehl et al., 2021; Nora et al., 2022; Zhu et al., 2024), and inclusion complexation (Munnangi et al., 2023; Oo et al., 2022; Schoeman et al., 2024; Tsunoda et al., 2023). Among these, the amorphous solid dispersion (ASD) method has become one of the most effective and extensively researched techniques to improve the solubility of poorly water-soluble drugs (Budiman and Aulifa, 2022; Koromili et al., 2023). ASDs can significantly improve dissolution rate and generate supersaturation in the gastrointestinal tract, by dispersing the drug in a polymeric matrix in its high-energy amorphous state (Polyzois et al., 2024; Yun et al., 2024). However, some of conventional binary ASDs, typically composed of a drug and a single polymer, have several limitations alongside their successes. These limitations include physical instability, low drug loading capacity, and inadequate recrystallization control, which may compromise long-term performance and limit their ability in industrial conversion (Budiman et al., 2024; Budiman et al., 2023b; Budiman et al., 2022; Newman et al., 2008).

To overcome the limitations of binary ASD systems, the pharmaceutical field has been looking for innovative ways to enhance the properties of these systems. One of the main strategies that have been explored is the use of ternary ASD systems. In ternary ASDs systems, the main components of the binary systems are incorporated with a third component, typically a surfactant, additional polymer, or small molecule stabilizer (Saberi et al., 2023; Tian et al., 2020). This approach has revealed significant potential to substantially increase kinetic and thermodynamic solubility of drugs, prevent drug recrystallization, and maintain supersaturation levels (Amaliah et al., 2025; Borde et al., 2021; Budiman et al., 2023a; Sohn et al., 2020). Mechanistic interactions between drug, polymer, and excipient components can result in synergistic effects that are not achievable in binary systems, such as micellar solubilization, hydrogen bonding stabilization, and nanocluster formation (Kosaka et al., 2020; Omagari et al., 2020; Pöstges et al., 2023; Zhao et al., 2019). As a result, ternary ASDs are rapidly emerging as a next-generation formulation approach with superior biopharmaceutical performance.

While several studies have investigated various ternary combinations and their effects on drug solubility, a systematic and quantitative synthesis of the available evidence remains absent. Existing literature is often fragmented—focusing on isolated case studies or limited formulation types—without offering a comparative framework that integrates formulation composition, mechanistic interaction, and solubility outcomes. To our knowledge, no comprehensive comparative evaluation has been conducted to determine which specific type of ternary ASD formulation—such as drug–polymer–polymer, drug–polymer–Surfactant, drug–polymer–small molecule or drug–drug–polymer—is most effective in improving drug solubility across diverse pharmaceutical compounds.

This review addresses this critical gap by presenting a scientifically rigorous and quantitatively grounded comparative assessment of ternary ASD systems, with a focus on solubility enhancement. Through comparative evaluation of peer-reviewed studies, we classify ternary systems based on their compositional design and mechanistic functionality, analyze their relative performance in solubility improvement, and identify key formulation principles driving their success. This review not only provides the first evidence-based ranking of ternary ASD strategies, but also offers mechanistic insights and practical guidance for rational formulation design.

Accordingly, the aim of this review is to critically evaluate and compare the solubility-enhancing efficacy of various ternary ASD systems, highlight the underlying molecular mechanisms responsible for their performance, and identify the most promising formulation strategies. By combining a systematic comparative approach with mechanistic interpretation, this review aspires to advance the current understanding of ternary ASDs and support the development of more effective drug delivery systems for poorly soluble compounds.

## 2 Methodology

**Fig. 1:**
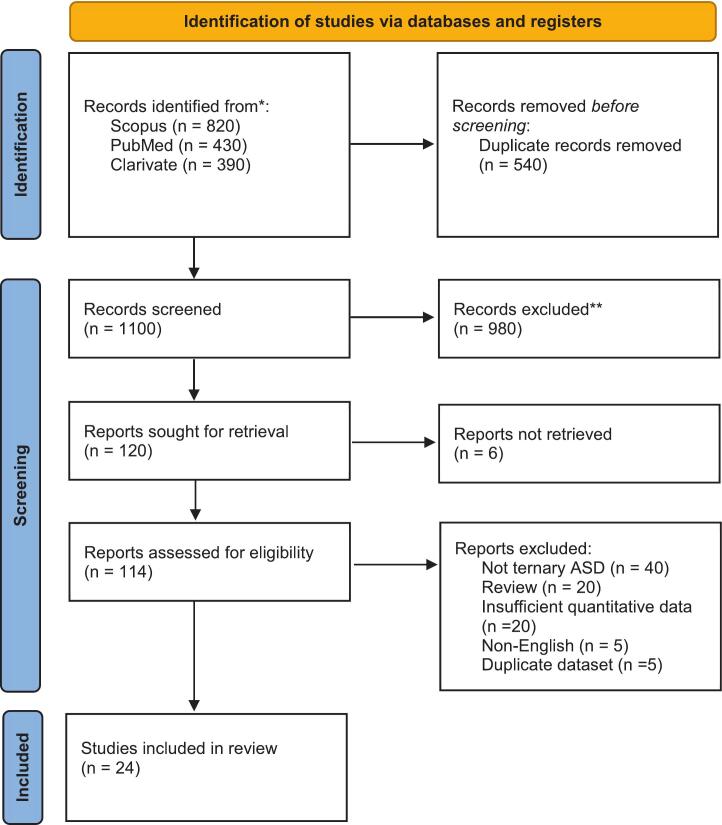
Flowchart illustrating the identification and selection of studies.

This review adopts a systematic approach to investigate the ternary system based on amorphous solid dispersion (ASD), specifically focusing on the intricate interactions within the binary system of amorphous drug and the critical role of surfactants. Our structured approach begins with a comprehensive literature search across esteemed databases—PubMed, Clarivate, and Scopus—encompassing publications since 2015. This search included precise keywords like “ASD Ternary,” “Amorphous Solid Dispersion Ternary,” “Drug:Polymer,” and “Drug Polymer” to ensure the selection of peer-reviewed papers, original research, and high-quality reviews. The selected studies were evaluated according to predefined inclusion and exclusion criteria, and relevant data were extracted to examine the preparation, characterization, and performance of TSD systems. Quantitative information on solubility enhancement was compiled to allow comparative evaluation of ternary system. Where applicable, trends in the performance of polymers, surfactants, and small-molecule excipients were examined to identify consistent patterns and formulation-dependent variability. Fig. 1 presents a flow diagram summarizing the study selection process. The review underscores the physicochemical advantages of ternary systems, highlights existing limitations, and proposes directions for future research. (See Table 1, Table 2, Table 3.)

**Table 1:** Current development of ternary system based on amorphous solid dispersion.

Ternary System	API and The Main System	Method Preparation	Pure Drug Solubility μg/mL	Ternary System Solubility μg/mL	Improvement (Fold)	Ref
Drug:Polymer:Polymer	Abirateron: kinetisol (HPBCD): Eudragit L100–55	Solvent evaporation	11.000	45.000	4	(Gala et al., 2020)
Drug:Polymer:Polymer	Abirateron: kinetisol (HPBCD): HPMCAS 716 G	Solvent evaporation	11.000	40.000	4	(Gala et al., 2020)
Drug:Polymer:Polymer	Abirateron: kinetisol (HPBCD): HPMCAS126G	Solvent evaporation	11.000	82.000	7	(Gala et al., 2020)
Drug:Polymer:Polymer	Abirateron: kinetisol (HPBCD): HPMCAS912G	Solvent evaporation	11.000	66.000	6	(Gala et al., 2020)
Drug:Polymer:Polymer	Abirateron: kinetisol (HPBCD): HPMCE15	Solvent evaporation	11.000	68.000	6	(Gala et al., 2020)
Drug:Polymer:Polymer	Abirateron: kinetisol (HPBCD): HPMCE50	Solvent evaporation	11.000	73.000	7	(Gala et al., 2020)
Drug:Polymer:Polymer	Abirateron: kinetisol (HPBCD): NACMC	Solvent evaporation	11.330	39.000	3	(Gala et al., 2020)
Drug:Polymer:Polymer	Abirateron: kinetisol (HPBCD): PVPAP	Solvent evaporation	11.330	39.000	3	(Gala et al., 2020)
Drug:Polymer:Polymer	Abirateron: kinetisol (HPBCD): PVPK90	Solvent evaporation	11.000	53.000	5	(Gala et al., 2020)
Drug:Polymer:Polymer	Atorvastatin: PS630: PEG 400	Hot Melt Extrusion	16.000	40.000	3	(Sarabu et al., 2022)
Drug:Polymer:Polymer	Atorvastatin: PS630U: PEG400	Hot Melt Extrusion	16.000	40.000	3	(Sarabu et al., 2022)
Drug:Polymer:Polymer	Celecoxib (10 %): MA-EA: HPC	hot melt extrusion	3.000	160.000	53	(Pöstges et al., 2022)
Drug:Polymer:Polymer	Curcumin: HPMC E5: Poloxamer 188 (F4-F6)	solvent evaporation	2.400	78.000	33	(Ishtiaq et al., 2024)
Drug:Polymer:Polymer	Curcumin: HPMC E5: Solupus (F1-F3)	solvent evaporation	2.400	186.000	78	(Ishtiaq et al., 2024)
Drug:Polymer:Polymer	Curcumin: HPMC E5: Syloid 244 (F10-F12)	solvent evaporation	2.400	60.000	25	(Ishtiaq et al., 2024)
Drug:Polymer:Polymer	Curcumin: HPMC E5: Syloid XDP3150 (F7-F9)	solvent evaporation	2.400	63.000	26	(Ishtiaq et al., 2024)
Drug:Polymer:Polymer	Indometacin: Eudragit EPO: PVPK30	Hot Melt Extrusion	1.500	15.000	10	(Wang et al., 2020)
Drug:Polymer:Polymer	Indometacin: HPMC: Mesoporous Silica	Hot Melt Extrusion	0.940	20.000	21	(Hanada et al., 2018b)
Drug:Polymer:Polymer	Indometacin: PVPVA: PEO	Hot-Melt Extrusion	0.940	7.500	8	(Pezzoli et al., 2019)
Drug:Polymer:Polymer	Itraconazole: HPMCAS: Poloxamer 188	hot-melt extrusion	0.960	20.000	21	(Solanki et al., 2019)
Drug:Polymer:Polymer	Itraconazole: HPMCAS: Poloxamer 407	hot-melt extrusion	0.960	40.000	41	(Solanki et al., 2019)
Drug:Polymer:Polymer	Itraconazole: PovidoneK12: Povidon K12	Hot Melt Extrusion	1.200	7.000	6	(Meng et al., 2017)
Drug:Polymer:Polymer	Nimodipin (10–30 %): HPMCE5: Eudragit	Hot-Melt Extrusion	0.030	20.000	667	(Hörmann et al., 2018)
Drug:Polymer:Polymer	Regorfanib: HPMCAS: Povidone	solvent evaporation	0.600	37.000	62	(Müller et al., 2021)
Drug:Polymer:Polymer	Regorfanib: Povidone: HPMCAS	solvent evaporation	0.600	37.000	62	(Müller et al., 2021)
Drug:Polymer:Surfactant	Amphotericin B: HPMCAS 912: Sodium Deodecyl Sulfate (125)	Spray-drying (Via LAP)	0.580	97.940	169	(Smith-Craven et al., 2024)
Drug:Polymer:Surfactant	Atorvastatin: PS530U: Tween 80	Hot Melt Extrusion	16.000	40.000	3	(Sarabu et al., 2022)
Drug:Polymer:Surfactant	Atorvastatin: PS630: Tween 80	Hot-melt extrusion	16.000	40.000	3	(Sarabu et al., 2022)
Drug:Polymer:Surfactant	Bicalutamide: Copovidone: SDS	solvent evaporation	4.300	28.500	7	(Moseson et al., 2023)
Drug:Polymer:Surfactant	Bicalutamide: Copovidone: Vit E TPGS	solvent evaporation	4.300	28.000	7	(Moseson et al., 2023)
Drug:Polymer:Surfactant	Felodipine: PVPVA: DATPEGS	cryo-milled	19.700	160.000	8	(Saboo et al., 2021)
Drug:Polymer:Surfactant	Felodipine: PVPVA:TPGS	Solvent Shift Method	1.300	160.000	123	(Saboo et al., 2021)
Drug:Polymer:Surfactant	griseofulvin:HPC: Sodium dodecyl sulfate (SDS)	Hot Melt Extrusion and Spray Drying	8.640	18.300	2	(Rahman et al., 2020)
Drug:Polymer:Surfactant	griseofulvin:Soluplus: Sodium dodecyl sulfate (SDS)	Hot Melt Extrusion and Spray Drying	8.640	17.800	2	(Rahman et al., 2020)
Drug:Polymer:Surfactant	Itraconazole: HPMCAS: DATPEGS	hot-melt extrusion	0.960	340.000	353	(Solanki et al., 2019)
Drug:Polymer:Surfactant	Itraconazole: Eudragit: TPGS	spray dryer	0.050	3.981	80	(Deshpande et al., 2018)
Drug:Polymer:Surfactant	itraconazole: HPMCAS-HF: TPGS	spray dryer	0.050	10.688	214	(Deshpande et al., 2018)
Drug:Polymer:Surfactant	Itraconazole: PVP-VA: SLS	spray dryer	0.050	0.882	18	(Deshpande et al., 2018)
Drug:Polymer:Surfactant	Itraconazole: Soluplus: SLS	spray dryer	0.050	9.980	200	(Deshpande et al., 2018)
Drug:Polymer:Surfactant	L-tetrahydropalmatine:Hydroxypropyl Methylcellulose Phthalate:Poloxamer 188	Solvent Shift Method	2.800	4.116	1	(Tung et al., 2021)
Drug:Polymer:Surfactant	Ritonavir (30 %): PVPVA: SDS	solvent evaporation	0.370	210.000	568	(Yang et al., 2023)
Drug:Polymer:Surfactant	Ritonavir (30 %): PVPVA: Span	solvent evaporation	0.370	300.000	811	(Yang et al., 2023)
Drug:Polymer:Surfactant	Ritonavir (30 %): PVPVA: Tween	solvent evaporation	0.370	105.000	284	(Yang et al., 2023)
Drug:Polymer:Surfactant	Ritonavir: PVPVA: Span 20	Hot-Melt Twin Screw Extruder	5.000	111.100	22	(Wu et al., 2023)
Drug:Polymer:Surfactant	Ritonavir: Polyvinylpyrrolidone vinyl acetate: Poloxamer 407	Film Casting Method	2.200	35.000	16	(Siriwannakij et al., 2021)
Drug:Polymer:Surfactant	Ritonavir: Polyvinylpyrrolidone vinyl acetate: Span 20	Film Casting Methode	2.200	35.000	16	(Siriwannakij et al., 2021)
Drug:Polymer:Excipient	Fenofibrate (20 %): PVPVA: HPL	freeze-drying	1.330	100.000	75	(Czajkowski et al., 2023)
Drug:Polymer:Excipient	Fenofibrate (20 %): PVPVA: HPL	freeze-drying	4.720	100.000	21	(Czajkowski et al., 2023)
Drug:Polymer:Excipient	Fisetin (30 %): Eudragit (EL100): HP- β-cyclodextrin	cryogenic ball milling	2.030	318.130	157	(Rosiak et al., 2024)
Drug:Polymer:Excipient	Fisetin (30 %): Eudragit (EPO): HP- β-cyclodextrin	cryogenic ball milling	2.030	126.500	62	(Rosiak et al., 2024)
Drug:Polymer:Excipient	Ritonavir: Copovidon: Colloidal silicon dioxide (Ph 1)	Melt Method	570.000	960.000	2	(Rosiak et al., 2024)
Drug:Polymer:Excipient	Ritonavir: Copovidon: Colloidal silicon dioxide (Ph 2)	Melt Method	10.000	310.000	31	(Njoku et al., 2020)
Drug:Polymer:Excipient	Ritonavir: Copovidon: Colloidal silicon dioxide (Ph 6,8)	Melt Method	2.000	60.000	30	(Njoku et al., 2020)
Drug:Drug:Polymer	sulfamethoxazole (SMZ): trimethoprim (TMP): Eudragit EPO	Melt Quenching	–	–	6.4	(Li et al., 2022)
Drug:Drug:Polymer	sulfamethoxazole (SMZ): trimethoprim (TMP): Polyacrylic acid	Melt Quenching	–	–	4.6	(Li et al., 2022)
Drug:Drug:Polymer	ezetimibe (EZE): lovastatin (LOV): Soluplus®	Spray Drying	0.900 (EZE) and 3.800 (LOV)	26.000 (EZE) and 3.100 (LOV)	27 (EZE)	(Riekes et al., 2016)
Drug:Drug:Polymer	ritonavir (RTV): lopinavir (LPV): PVP	Solvent Evaporation	1.700 (RTV) and 2.700 (LPV)	9.000 (RTV) and 8.000 (LPV)	5.29 (EZE) and 2.96 (LPV)	(Trasi and Taylor, 2015)
Drug:Drug:Polymer	ritonavir (RTV): lopinavir (LPV): HPMCAS	Solvent Evaporation	1.700 (RTV) and 2.700 (LPV)	11.400 (RTV) and 9.100 (LPV)	6.7 (EZE) and 3.37 (LPV)	(Trasi and Taylor, 2015)
Drug:Drug:Polymer	flutamide (FL): bicalutamide (BIC): PVP	Hot Plate Melting	13.400 (FL) and 1.400 (BIC)	27.200 (FL) and 9.900 (BIC)	2 (FL) and 7 (BIC)	(Pacult et al., 2019)
Drug:Drug:Polymer	cefdinir (CEF): curcumin (CUR): PVP	Solvent Evaporation	1.765 (CEF)	4.701 (CEF)	2.663 (CEF)	(Naama et al., 2025)

**Table 2:** The impact of ternary system amorphous solid dispersion on release profile.

Ternary System	API and The Main System	Improvement	Dissolution and Release Behavior	Ref
Drug: Polymer: Polymer	Atorvastatin: PS630: PEG 400	2.500	>90 % after 60 min (highest) F6 (PEG400): DE60 = 90.23, IDR = 6.79, MDR = 2.28; F2: DE60 = 88.28; Pure ACT: DE60 = 33.16, IDR = 1.15	(Sarabu et al., 2022)
Drug: Polymer: Polymer	Atorvastatin: PS630U: PEG400	2.500	>90 % after 60 min	(Sarabu et al., 2022)
Drug: Polymer: Polymer	Itraconazole: PovidoneK12: Povidon K12	5.830	7 μg/mL (supersaturation 6×)	(Meng et al., 2017)
Drug: Polymer: Polymer	Abirateron: kinetisol (HPBCD): HPMCAS126G	7.450	60 μg/mL after 90 min (The study demonstrates that the ternary ASD of abiraterone:HPBCD:HPMCAS 126G (10:80:10 *w*/w) achieved the highest dissolution and supersaturation compared to both the binary system (abiraterone:HPBCD) and other ternary variants, highlighting the critical role of HPMCAS 126G inclusion and optimal molar ratio in enhancing abiraterone release.	(Gala et al., 2020)
Drug: Polymer: Polymer	Indometacin: PVPVA: PEO	8.000	∼80 % after 75 min (Invitro: 7.5 % IND: >80 % after 75 min 15 % & 30 % IND: Doesn't dissolve, tablet form remains intact (pH 1.2), all formulation >80 % after 130 menit (pH 6.8)	(Pezzoli et al., 2019)
Drug: Polymer: Polymer	Indometacin: Eudragit EPO: PVPK30	10.000	<6 μg/mL	(Wang et al., 2020)
Drug: Polymer: Polymer	Itraconazole: HPMCAS: Poloxamer 188	20.750	10 % after 2 h	(Solanki et al., 2019)
Drug: Polymer: Polymer	Indometacin: HPMC: Mesoporous Silica	21.340	217.5 μg/mL (High-SME samples (2- and 3-kneading zones): Maintained supersaturation for up to 24 h → strong “parachute effect” → superior in vitro dissolution behavior)	(Hanada et al., 2018b)
Drug: Polymer: Polymer	Itraconazole: HPMCAS: Poloxamer 407	41.490	40 % after 2 h	(Solanki et al., 2019)
Drug: Polymer: Polymer	Celecoxib (10 %): MA-EA: HPC	53.330	10 % after 80 min	(Pöstges et al., 2022)
Drug: Polymer: Polymer	Regorfanib: HPMCAS: Povidone	61.670	(<10 μg/mL at 90 min) (In vitro Gastric simulation (FaSSGF): RGF_PVP: after 120 min in acidic medium, release dropped to ∼4 μg/mL.RGF_HPMCAS: unaffected, showing resistance to gastric fluid. Co-administered HPMCAS: not sufficient to protect against gastric fluid effects. In vivo AUC: 166.8 → 241.5 mg·h/L (not statistically significant, *p* = 0.34) tmax: 2.25 vs 2.5 h. High inter-individual variability in both groups)	(Müller et al., 2021)
Drug: Polymer: Polymer	Regorfanib: Povidone: HPMCAS	61.670	(<10 μg/mL at 90 min) (In vitro Gastric simulation (FaSSGF): RGF_PVP: after 120 min in acidic medium, release dropped to ∼4 μg/mL.RGF_HPMCAS: unaffected, showing resistance to gastric fluid. Co-administered HPMCAS: not sufficient to protect against gastric fluid effects. In vivo AUC: 166.8 → 241.5 mg·h/L (not statistically significant, p = 0.34) tmax: 2.25 vs 2.5 h. High inter-individual variability in both groups)	(Müller et al., 2021)
Drug: Polymer: Polymer	Curcumin: HPMC E5: Solupus (F1-F3)	77.500	F3 formulation, containing 20 % Soluplus®, achieved the highest dissolution rate: 91 % ± 3.89 %, compared to pure curcumin which exhibited only 10 % ± 2.58 % dissolution.	(Ishtiaq et al., 2024)
Drug: Polymer: Polymer	Nimodipin (10–30 %): HPMCE5: Eudragit	666.670	>85 % after 30 min (In vitro: >85 % API dissolved in 30 min (immediate release target)	(Hörmann et al., 2018)
Drug: Polymer: Surfactant	L-tetrahydropalmatine:Hydroxypropyl Methylcellulose Phthalate:Poloxamer 188	1.470	<50 %	(Tung et al., 2021)
Drug: Polymer: Surfactant	griseofulvin:Soluplus: Sodium dodecyl sulfate (SDS)	2.060	Ternary HyNASD formulations composed of GF:Sol:SDS at 1:5:0.05 ratios achieved exceptionally high GF supersaturation—up to 300 % within 20 min, surpassing both physical mixtures and traditional nanocomposites. In contrast, binary systems or formulations without SDS failed to exceed ∼50 % supersaturation, underscoring the critical role of SDS for wettability and Sol polymer for recrystallization inhibition	(Rahman et al., 2020)
Drug: Polymer: Surfactant	griseofulvin:HPC: Sodium dodecyl sulfate (SDS)	2.120	Ternary HyNASD formulations composed of GF:Sol:SDS at 1:5:0.05 ratios achieved exceptionally high GF supersaturation—up to 300 % within 20 min, surpassing both physical mixtures and traditional nanocomposites. In contrast, binary systems or formulations without SDS failed to exceed ∼50 % supersaturation, underscoring the critical role of SDS for wettability and Sol polymer for recrystallization inhibition	(Rahman et al., 2020)
Drug: Polymer: Surfactant	Atorvastatin: PS530U: Tween 80	2.500	>90 % after 60 min	(Sarabu et al., 2022)
Drug: Polymer: Surfactant	Atorvastatin: PS630: Tween 80	2.500	>90 % after 60 min	(Sarabu et al., 2022)
Drug: Polymer: Surfactant	Bicalutamide: Copovidone: Vit E TPGS	6.510	Ternary ASDs with 1.5–3 % TPGS showed slightly faster BCL release and higher supersaturation than binary systems, but cryomilling reduced performance (∼18 μg/mL).	(Moseson et al., 2023)
Drug: Polymer: Surfactant	Bicalutamide: Copovidone: SDS	6.630	Formula with 3 % SDS maintained fast, congruent release even after cryomilling, indicating greater formulation resilience.	(Moseson et al., 2023)
Drug: Polymer: Surfactant	Felodipine: PVPVA: DATPEGS	8.120	0.001 mg/min/cm2	(Saboo et al., 2021)
Drug: Polymer: Surfactant	Ritonavir: Polyvinylpyrrolidone vinyl acetate: Poloxamer 407	15.910	77 % release in 75 min (pH 2.0), ∼9 % release in 15 min (pH 6.8), 65 % dissolution in 60 min (FeSSIF-V2)	(Siriwannakij et al., 2021)
Drug: Polymer: Surfactant	Ritonavir: Polyvinylpyrrolidone vinyl acetate: Span 20	15.910	47 % release at 75 min (pH 2.0) ∼9 % release in 15 min (pH 6.8), 71 % dissolution in 120 min (FeSSIF-V2)	(Siriwannakij et al., 2021)
Drug: Polymer: Surfactant	Itraconazole: PVP-VA: SLS	17.640	4 μg/mL up to 60 min	(Deshpande et al., 2018)
Drug: Polymer: Surfactant	Ritonavir: PVPVA: Span 20	22.220	>90 % after 1 h	(Wu et al., 2023)
Drug: Polymer: Surfactant	Itraconazole: Eudragit: TPGS	79.620	4 μg/mL up to 60 min	(Deshpande et al., 2018)
Drug: Polymer: Surfactant	Felodipine: PVPVA:TPGS	123.080	>55 % Release at 60 min	(Saboo et al., 2021)
Drug: Polymer: Surfactant	Amphotericin B: HPMCAS 912: Sodium Deodecyl Sulfate (125)	168.860	ternary ASDs (AmB + Surfactant + polymer) increased the dissolution concentration of Amphotericin B (AmB) by up to 90-fold, whereas binary ASDs (AmB + Surfactant only) achieved about a 40-fold increase, despite using 7.5× more Surfactant than the ternary system	(Smith-Craven et al., 2024)
Drug: Polymer: Surfactant	Itraconazole: Soluplus: SLS	199.600	4 μg/mL up to 60 min	(Deshpande et al., 2018)
Drug: Polymer: Surfactant	itraconazole: HPMCAS-HF: TPGS	213.760	4 μg/mL up to 60 min	(Deshpande et al., 2018)
Drug: Polymer: Surfactant	Ritonavir (30 %): PVPVA: Tween	283.780	6 mg/min/cm2	(Yang et al., 2023)
Drug: Polymer: Surfactant	Itraconazole: HPMCAS: DATPEGS	352.700	20 % after 2 h	(Solanki et al., 2019)
Drug: Polymer: Surfactant	Ritonavir (30 %): PVPVA: SDS	567.570	7 mg/min/cm2	(Yang et al., 2023)
Drug: Polymer: Surfactant	Ritonavir (30 %): PVPVA: Span	810.810	4 mg/min/cm2	(Yang et al., 2023)
Drug: Poymer: Excipient	Ritonavir: Copovidon: Colloidal silicon dioxide (Ph 1)	1.680	>60 % release in 60 min (pH 1.0)	(Njoku et al., 2020)
Drug: Poymer: Excipient	Fenofibrate (20 %): PVPVA: HPL	21.190	in FeSSIF medium: Dissolution profiles of FEN in all formulations showed higher initial drug concentrations (supersaturation) ∼100 μg/mL followed by gradually decreasing drug concentrations at 60 min reflecting recrystaliztion	(Czajkowski et al., 2023)
Drug: Poymer: Excipient	Ritonavir: Copovidon: Colloidal silicon dioxide (Ph 6,8)	30.000	>30 % dissolution in 60 min (pH 6.8)	(Njoku et al., 2020)
Drug: Poymer: Excipient	Ritonavir: Copovidon: Colloidal silicon dioxide (Ph 2)	31.000	>50 % release in 60 min (pH 2.0)	(Njoku et al., 2020)
Drug: Poymer: Excipient	Fenofibrate (20 %): PVPVA: HPL	75.190	in FaSSIF medium: Dissolution profiles of FEN in all formulations showed higher initial drug concentrations (supersaturation) ∼100 μg/mL followed by gradually decreasing drug concentrations at 60 min reflecting recrystaliztion	(Czajkowski et al., 2023)
Drug: Polymer: Surfactant	Ritonavir: Polyvinylpyrrolidone/vinyl acetate:Sodium dodecyl sulfate (30 %DL)	–	>70 % Release at 30 min	(Yang et al., 2023)
Drug: Polymer: Surfactant	Ritonavir: Polyvinylpyrrolidone/vinyl acetate:Span 20 (30 %DL)	–	100 % Release at 45 min	(Yang et al., 2023)
Drug: Polymer: Surfactant	Ritonavir: Polyvinylpyrrolidone/vinyl acetate:Span 85 (30 %DL)	–	100 % Release at 45 min	(Yang et al., 2023)
Drug: Polymer: Surfactant	Ritonavir: Polyvinylpyrrolidone/vinyl acetate:Tween 80 (30 %DL)	–	>50 % Release at 90 min	(Yang et al., 2023)
Drug: Polymer: Surfactant	Ritonavir: PVPVA: SLS	–	80 % at 90 min	(Indulkar et al., 2022)
Drug: Polymer: Surfactant	Ritonavir: PVPVA: span 20	–	40 % at 90 min	(Indulkar et al., 2022)
Drug: Polymer: Surfactant	Ritonavir: PVPVA: span 85	–	90 % at 90 min	(Indulkar et al., 2022)
Drug: Polymer: Surfactant	Ritonavir: PVPVA: TPGS	–	90 % at 90 min	(Indulkar et al., 2022)
Drug: Polymer: Surfactant	Ritonavir: PVPVA: tween 80	–	30 % at 90 min	(Indulkar et al., 2022)
Drug: Drug: Polymer	Sulfamethoxazole (SMZ): trimethoprim (TMP): Eudragit EPO	6.400-fold for SMZ and 1.9-fold for TMP	80 % at 10 min	(Li et al., 2022)
Drug: Drug: Polymer	sulfamethoxazole: trimethoprim: Polyacrylic acid (PAA)	2.600-fold for SMZ and 4.600-fold for TMP	80 % at 10 min	(Li et al., 2022)
Drug: Drug: Polymer	ezetimibe (EZE): lovastatin (LOV): Soluplus®	18.000-fold for EZE and 6.000-fold for LOV	92 % of EZE and 83 % of LOV dissolved within 5 min.	(Riekes et al., 2016)
Drug: Drug: Polymer	ritonavir (RTV): lopinavir (LPV): PVP	3.000	60 % at 20 min.	(Trasi and Taylor, 2015)
Drug: Drug: Polymer	ritonavir (RTV): lopinavir (LPV): HPMCAS	3.000	60 % at 20 min.	(Trasi and Taylor, 2015)
Drug: Drug: Polymer	flutamide (FL): bicalutamide (BIC): PVP	2.000-fold for FL and 3.000-fold for BIC	25 % of FL and 5 % of BIC dissolved after 60 min.	(Pacult et al., 2019)
Drug: Drug: Polymer	cefdinir (CEF): curcumin (CUR): PVP	2.250	90 % of CEF in 30 min.	(Naama et al., 2025)

**Table 3:** The impact of ternary system amorphous solid dispersion on drug bioactivity and pharmacological properties.

Ternary System	API and The Main System	Improvement	Invitro-Invivo Effectivity Study	Ref
Drug: Polymer: Polymer	Abirateron: kinetisol (HPBCD): HPMCAS126G	7.450	A pharmacokinetic study revealed that HPBCD based binary and ternary KSDs enhanced abiraterone bioavailability by 12.4-fold, and 13.8-fold, respectively, compared to a generic abiraterone acetate tablet.	(Gala et al., 2020)
Drug: Polymer: Polymer	Curcumin: HPMC E5: Solupus (F1-F3)	77.500	Biological testing of F3 also exhibited superior anti-bacterial, anti-oxidant and anti-inflammatory activities in comparison to pure curcumin suggesting F3 to be an effective vehicle in delivering curcumin.	(Ishtiaq et al., 2024)
Drug: Polymer: Polymer	Indometacin: Eudragit EPO: PVPK30	10.000	In vitro: At 2 μg/mL: Only 64 % precipitated (significant inhibition). At 50 μg/mL: 27.6 % precipitated (excellent inhibition). Higher concentrations (1 mg/mL) did not further improve inhibition. Invivo:-	(Wang et al., 2020)
Drug: Polymer: Surfactant	L-tetrahydropalmatine:Hydroxypropyl Methylcellulose Phthalate:Poloxamer 188	1.470	Binary and ternary ASDs markedly improved 1-THP exposure. Cmax rose from 14.620 ng/mL (pure) to 66.480 ng/mL (binary) and 45.59 ng/mL (ternary), while AUCINF,pred increased from 30.170 to 101.000 (binary) and 41.870 h·ng/mL (ternary). Bioavailability improved to 334.77 % (binary) and 138.78 % (ternary), confirming the strong pharmacokinetic advantage of ASD systems	(Tung et al., 2021)
Drug: Poymer: Excipient	Fenofibrate (20 %): PVPVA: HPL	75.190	in vitro permeation was found substantially enhanced (2.250 μg final mass permeated)as compared to a suspension of crystalline FEN and at least equal compared to marketed formulations under comparable conditions	(Czajkowski et al., 2023)
Drug: Poymer: Excipient	Fenofibrate (20 %): PVPVA: HPL	21.190	in vitro permeation was found substantially enhanced (2.250 μg final mass permeated)as compared to a suspension of crystalline FEN and at least equal compared to marketed formulations under comparable conditions	(Czajkowski et al., 2023)
Drug: Poymer: Excipient	Fisetin (30 %): Eudragit (EL100): HP- β-cyclodextrin	156.710	Among all formulations, ASI_30_EPO displayed the most balanced and superior antioxidant and neuroprotective profile, suggesting the synergistic benefit of 30 % loading with Eudragit EPO and HPβCD in enhancing fisetin's bioactivity.	(Rosiak et al., 2024)
Drug: Poymer: Excipient	Fisetin (30 %): Eudragit (EPO): HP- β-cyclodextrin	62.320	Among all formulations, ASI_30_EPO displayed the most balanced and superior antioxidant and neuroprotective profile, suggesting the synergistic benefit of 30 % loading with Eudragit EPO and HPβCD in enhancing fisetin's bioactivity.	(Rosiak et al., 2024)
Drug: Drug: Polymer	cefdinir (CEF): curcumin (CUR): PVP	1.833	The ternary ASD demonstrated a synergistic effect in antibacterial efficacy, as indicated by greater inhibition zones against *Staphylococcus aureus* (50 mm) and *Proteus vulgaris* (55 mm) in contrast to pure CEF, which displayed inhibition zones of 35 mm and 30 mm, respectively.	(Naama et al., 2025)

## 3 Amorphous solid dispersion

### 3.1 Basic concept of amorphous solid dispersion

Amorphous solid dispersions (ASDs) are widely employed to increase the solubility, dissolution rate, and bioavailability of poorly soluble drugs, mainly for BCS Class II and IV drugs (Tambe et al., 2022). The core principle of ASDs involves dispersing the active pharmaceutical ingredient (API) in an amorphous form into a polymeric matrix and consequently prevent recrystallization and enhance dissolution performance (Cao et al., 2023; Zhang et al., 2023).

ASDs enhance drug absorption by forming a supersaturated solution upon dissolution, in which the amorphous form achieves an initial burst of drug (spring) while polymer prevents rapid precipitation (parachute) through molecular interactions such as hydrogen bonding, van der Waals forces, and hydrophobic interactions (Tambe et al., 2022). Since the amorphous state is inherently unstable and tends to recrystallization, adding polymeric carriers is essential to inhibit crystallization via these interactions (Rosiak et al., 2022).

For instance, the ASD of hesperidin formulated with Soluplus® and HPMC showed significant improvements in solubility, attributed to hydrogen bonding between hesperidin and the polymer matrices, as well as the polymer's ability to elevate the glass transition temperature (Tg) of the system (Rosiak et al., 2022). Similarly, in the case of lumefantrine ASDs, polymers like PVPVA and enteric polymers such as hypromellose acetate succinate (HPMCAS) not only enhanced dissolution but also modulated drug release kinetics based on drug loading levels and polymer-drug interactions (Hiew et al., 2022). While PVPVA facilitated congruent release at low drug loads due to its hydrophilic-lipophilic balance, enteric polymers such as Eudragit L100 provided better physical stability at the expense of slower release rates (Hiew et al., 2022). Overall, ASD technology is fundamentally reliant on the rational selection of polymers, which function as crystallization inhibitors, release modulators, and stabilizers that influence the dissolving characteristics and bioavailability of the drug product (Tambe et al., 2022; Zhang et al., 2023).

### 3.2 Preparation technique of amorphous solid dispersion

The preparation of amorphous solid dispersions (ASDs) is an essential step for improving the solubility and bioavailability of poorly water-soluble drugs, with several studies emphasizing the necessity of selecting a suitable preparation method according to the properties of the drug and polymer. Hot-melt extrusion (HME) is a leading technique owing to its solvent-free process and scalability, as evidenced by Alzahrani et al. (2022), who emphasized its efficiency in producing physically stable ASDs while reducing time to market through thermodynamic and miscibility predictions. Wilke et al. (2024) assessed numerous techniques, including melt quenching, rotary evaporation, spray drying, and acoustic levitation, for the preparation of ketoprofen/PVP ASDs, demonstrating that each method distinctly affects the molecular structure and stability of the dispersions. For example, spray drying and melt quenching technologies demonstrate superior performance in stabilizing amorphous structures by mixing molecules more effectively than acoustic suspension techniques. Spray drying is a versatile method highly suitable for thermolabile drugs. Butreddy (2022) explored hydroxypropyl methylcellulose acetate succinate (HPMCAS)-based ASD prepared through spray drying and hot extrusion. The research proved that spray-dried particles were smaller and dissolved faster than HME, but both methods gave similar in vivo bioavailability, which was due to the formation of nanoparticles upon dissolution. Jara et al. (2021) have also confirmed the roles of nanoparticles that result from ASD dissolution. The research showed that niclosamide ASD hot-melt with PVP–VA resulted in colloidal nanoparticles within biorelevant media, which greatly enhanced in vitro diffusion rates and in vivo bioavailability. Overall, such studies indicate the tremendous impact of preparation processes on the molecular structure, stability, dissolution behavior, and bioavailability of ASD. This means that no universal optimal method exists, and optimization strategies have to be formulated according to the nature of the drug-polymer system.

### 3.3 Characterization and evaluation of amorphous solid dispersion

Several analytical techniques have been employed to characterize and evaluate amorphous solid dispersions (ASDs) with the aim of understanding their dissolution behavior, molecular interactions, and physicochemical stability. These techniques are crucial for monitoring structural transformation and determining the effectiveness of ASDs in increasing drug solubility and bioavailability. For example, Jiang et al. (2021) investigated the amorphization of cyclosporine A-based ASDs by X-ray Powder Diffraction (XRPD), demonstrating a distinctive halo pattern that confirms the absence of crystallinity and successful transition to the amorphous phase. Dharani et al. (2022) similarly demonstrated drug–polymer miscibility via Differential Scanning Calorimetry (DSC), wherein a singular glass-transition temperature (Tg) signifies a homogeneous molecular dispersion. Ravikumar et al. (2022) employed Fourier Transform Infrared Spectroscopy (FT-IR) to observe a significant shift in the carvedilol hydrogen-bond peak, confirming strong drug–polymer interactions.

Beyond solid-state characterization, solubility and precipitation studies provide complementary insights. Koromili et al. (2023) analyzed precipitation inhibition by surface-normalized release studies, demonstrating that PVP-based ASDs maintain sustained supersaturation, hence significantly improving dissolution rates in simulated saliva. Mehenni et al. (2018) demonstrated that selecting appropriate polymers enhances thermodynamic stability, as evidenced by elevated Tg values observed via DSC and XRPD during accelerated storage. Wang et al. (2024) confirmed the efficacy of polymers through dissolution studies, showcasing that ASDs composed of Soluplus and HPMCAS result in substantial enhancement in bioavailability by maintaining supersaturation in gastrointestinal fluids. Barmpalexis et al. (2019) conducted stability assessments using high-temperature storage studies, demonstrating that high molecular-weight polymers significantly hinder molecular mobility, hence delaying recrystallization in long-term storage. Lahiani-Skiba et al. (2011) compared solid-state transitions using FT-IR and XRPD, highlighting hydrogen bonds as critical for stabilizing amorphous formulations. Further surface analysis corroborated this effect. Zhang et al. (2019) correlated dissolution performance with surface-morphology analysis, wherein scanning electron microscopy (SEM) reveals structural distinctions between rapidly dissolving ASDs and their crystalline counterparts. Dielectric analysis by Stanković et al. (2015) indicated that stronger drug–polymer ionic bonds enhance molecular dispersion, improving solubility while prolonging stability.

In addition to these conventional approaches, advanced spectroscopic and imaging methods have increasingly been applied to deepen understanding of drug–polymer miscibility and molecular-scale interactions in ASDs. Steady-state fluorescence spectroscopy offers a rapid, non-destructive probe of nanoscale phase separation, detecting subtle shifts in the emission spectrum that reflect changes in polarity and hydrogen bonding within the microenvironment. Purohit et al. (2017) used this method to investigate itraconazole–HPMC ASDs, where emission-band shifts revealed domain-size variations dependent on processing: spray-dried samples exhibited finely dispersed domains (< 10 nm), whereas solvent-cast samples produced larger domains (> 30 nm). These fluorescence-derived indicators of nanoscale heterogeneity directly correlated with solid-state stability and dissolution behavior, providing valuable predictive insight during formulation optimization.

Complementary spatial information is obtained through confocal fluorescence microscopy (CFM), which enables direct visualization of phase separation and domain morphology in amorphous matrices under various environmental conditions. Using environment-sensitive dyes that differentially partition between drug- and polymer-rich phases, CFM provides three-dimensional mapping of miscibility at micrometer to near-nanoscale resolution. In the miconazole–PVPVA system, confocal microscopy revealed water-induced phase separation during exposure to high humidity and throughout dissolution, establishing a mechanistic link between moisture-triggered heterogeneity and reduced stability (Saboo and Taylor, 2017). Such in-situ imaging offers a dynamic perspective on how processing and environmental factors influence microstructure and drug release behavior.

At an even more fundamental level, solid-state nuclear magnetic resonance (ssNMR) has become indispensable for quantifying drug–polymer miscibility and for elucidating molecular interactions. By analyzing ^1H relaxation times (T₁, T₁ρ) and spin-diffusion behavior, ssNMR distinguishes homogeneous dispersions—where the API and polymer exhibit comparable relaxation parameters—from heterogeneous systems showing domain-specific discrepancies. These measurements yield nanoscale estimates of phase-separation domain sizes (typically 1–100 nm) and reveal the chemical nature of interactions such as hydrogen bonding, ionic pairing, π–π stacking, and hydrophobic contacts. Duan et al. (2020) applied this approach to correlate NMR-derived domain dimensions with fluorescence-based estimates, jointly confirming nanoscale heterogeneity in drug–polymer dispersions and validating the complementary power of these methods. Collectively, ssNMR provides a quantitative and mechanistic framework linking molecular packing and dynamics to the physical stability and dissolution performance of ASDs (Li et al., 2021; Saboo and Taylor, 2017).

Together, these advanced analytical methods—steady-state fluorescence spectroscopy, confocal fluorescence microscopy, and solid-state NMR—extend conventional thermal and spectroscopic characterization by offering molecular- and spatial-scale insights into miscibility, phase separation, and interaction dynamics. Their integration with classical approaches (DSC, PXRD, FTIR) enables a holistic evaluation of ASD performance, bridging structural understanding with macroscopic stability and bioavailability outcomes.

### 3.4 Limitation of binary system amorphous solid dispersion

Amorphous solid dispersion (ASD) in binary systems have long been employed to address the poor water solubility of many drug molecules by dispersing them in a polymeric matrix. This strategy typically enhances dissolution rate and helps maintain an amorphous state; however, it also possesses inherent limitations that may restrict its application in long-term pharmaceutical formulations. Sun et al. (2018) revealed through PXRD and DSC analysis that single-polymer ASDs rapidly crystallize under accelerated storage conditions, indicating instability. In another study, Liu and Guo (2021) highlight the limited dissolution sustainability of binary ASDs via surface-normalized release studies, demonstrating that single-polymer dispersions frequently fail to maintain supersaturation, resulting in precipitation within minutes of contact with aqueous media. LaFountaine et al. (2017) analyze mucoadhesive properties in binary ASDs through mucosal adhesion testing, indicating that although single-polymer matrices may enhance solubility, they are deficient in maintaining adherence and facilitating targeted drug delivery within the gastrointestinal tract. Zecevic et al. (2014) additionally confirmed that HPMC-AS binary ASDs prepared via hot extrusion exhibit drug precipitation during pH changes, thereby reducing bioavailability. Moreover, LaFountaine et al. (2017) illustrate the rapid phase separation in binary ASDs via FT-IR and PXRD analysis, indicating that weaker polymer-drug interactions in two-component systems result in instability and suboptimal mechanical properties. Overall, these findings emphasize that although binary ASD systems play an important role in enhancing solubility, their limitations in maintaining amorphization, dissolution, stability, and site-specific delivery necessitate the exploration of more robust ternary systems as alternatives.

## 4 Ternary system amorphous solid dispersion

### 4.1 Basic concept of ternary system amorphous solid dispersion

The development of amorphous solid dispersion (ASD) technology offers an effective approach for improving the solubility and bioavailability of poorly soluble drugs, particularly for compounds classified as classes II and IV in the biopharmaceutics classification system (BCS) (Saberi et al., 2023). Although binary ASDs, consisting of a drug dispersed in a polymer matrix, have demonstrated improved solubility and stability, challenges such as recrystallization and phase separation require additional optimization strategies (Pöstges et al., 2022). Therefore, the addition of a third component to binary ASDs has led to the formation of ternary ASDs, which exhibit enhanced solubility, increased physical stability, and sustained supersaturation (Budiman et al., 2023a). Ternary ASDs can be categorized based on the type of third component introduced, which can be an additional polymer, a Surfactant, or a small-molecule excipient. Drug:Polymer:Polymer systems exploit the synergistic effects between two polymers to enhance drug solubility and inhibit recrystallization, as demonstrated by the improved supersaturation kinetics of celecoxib when combined with a methacrylic acid-ethyl acrylate copolymer and hydroxypropyl cellulose (Pöstges et al., 2022). Alternatively, Drug:Polymer:Surfactant systems exploit Surfactant-mediated micellization and molecular interactions to increase wettability and maintain supersaturation. Studies of the ternary ASD candesartan cilexetil demonstrated that the addition of polyvinylpyrrolidone K25 with l-arginine significantly improved solubility while maintaining the amorphous form (Alatas et al., 2024). The Drug:Polymer:Small Molecule Excipient system incorporates low molecular weight co-forms, including amino acids or organic acids, to modify intermolecular interactions and thereby stabilize the amorphous phase. A notable example is curcumin, which exhibited a 300-fold increase in solubility when formulated with tryptophan in a ternary ASD system using supercritical fluid technology (Garbiec et al., 2025). Finally, Drug:Drug:Polymer integrates multiple active pharmaceutical ingredients (APIs) within a polymeric matrix to achieve synergistic enhancement in solubility, stability, and pharmacokinetic performance, which offers distinct advantages in combination therapies. The ternary ASD of ritonavir-darunavir complex with cyclodextrin improves the solubility and stability of ritonavir, resulting in enhanced oral bioavailability and pharmacological efficacy (Nguyen and Van Den Mooter, 2014). The mechanistic basis for the superiority of ternary ASD systems over binary ASD systems lies in their ability to optimize intermolecular interactions and modulate the physicochemical properties of drugs. Hydrogen bonds, π-π stacking, and ionic interactions are crucial for stabilizing ternary systems. For example, in indomethacin-based formulations, ternary ASDs effectively prevent precipitation and prolong supersaturation times compared to binary formulations (Budiman et al., 2023b; Budiman et al., 2023a). Furthermore, molecular mobility studies using differential scanning calorimetry (DSC) and powder X-ray diffraction (XRPD) revealed that ternary ASD exhibits a higher glass transition temperature (Tg), indicating improved stability against crystallization (Saberi et al., 2023). Overall, the fundamental principle of ternary ASDs highlights their significant advantages over binary ASDs in terms of improving solubility, stabilizing the amorphous state, and optimizing the dissolution profile. Case studies of various drug systems demonstrate their diverse applications and potential for drug development. The addition of a third component in ASDs signifies a possible approach to address solubility-related formulation challenges and advancing oral drug delivery systems.

### 4.2 Primary objective of ternary system amorphous solid dispersion development

The primary objective of developing ternary system amorphous solid dispersions (ASDs) is to improve the solubility, dissolution rate, and physical stability of poorly water-soluble drugs beyond what binary ASDs can achieve, thereby addressing critical pharmaceutical formulation issues (Saberi et al., 2023). Binary ASDs, while effective in enhancing drug dissolution, frequently encounter challenges like recrystallization, phase separation, and restricted miscibility between the drug and polymer, hence requiring the addition of a third component to optimize molecular interactions (Pöstges et al., 2022). The addition of a third component—such as an additional polymer, Surfactant, or small-molecule excipient—can provide an additional stabilization pathway, thereby enhancing drug-polymer interactions and maintaining supersaturation in aqueous media, ultimately improving bioavailability (Saberi et al., 2023). Several studies have demonstrated these benefits in practice Alatas et al. (2024) reported that the addition of l-arginine and polyvinylpyrrolidone K25 to a candesartan cilexetil formulation resulted in significantly higher solubility than the binary dispersions, as evidenced by an increase in glass transition temperature and a decrease in crystallinity in X-ray diffraction analysis. Similarly, Pöstges et al. (2022) showed that combining hydroxypropyl cellulose with a methacrylic acid–ethyl acrylate copolymer in celecoxib ASD promoted synergistic polymer interactions, resulting in enhanced solubility and prolonged supersaturation. Mechanistically, ternary ASDs function by utilizing various stabilization pathways, including hydrogen bonding, π–π interactions, and ionic complexation, which inhibit molecular mobility and obstruct recrystallization, as demonstrated in research on indomethacin ternary ASDs (Budiman et al., 2023a). Additionally, reported a significant 300-fold increase in curcumin solubility when tryptophan was combined with a polymer carrier using supercritical fluid technology (Garbiec et al., 2025). Surfactants have also proven to be effective; for example, sodium dodecyl sulfate maintains supersaturation and prevents precipitation in itraconazole ASD, resulting in a significantly improved solubility profile (Saberi et al., 2023). These findings collectively underscore that the advancement of ternary ASDs is driven by the need to address the limitations of binary formulations, hence providing enhanced amorphous stability, improved solubility, and better pharmacokinetic performance for poorly water-soluble drugs.

### 4.3 Advantages of ternary system amorphous solid dispersion

Ternary amorphous solid dispersions (TSDs) are increasingly recognized as a viable means of addressing the obstacle presented by water-insolubility of drugs (Shen et al., 2024). In contrast to conventional binary dispersions composed entirely of drug and polymer, TSDs incorporate a third excipient in the form of a small molecule, surfactant, or secondary polymer providing additional stability and functionality advantages (Alhayali et al., 2016; Amaliah et al., 2025; Budiman et al., 2023a; Prasad et al., 2016). This supplementary component plays a key role in avoiding recrystallization, maintaining supersaturation, and increasing the physical stability of the formulation (Guan et al., 2019). Several examples illustrate these advantages. Hanada et al., 2018a, Hanada et al., 2018b have reported that the addition of a porous carrier to indomethacin-based dispersion enhanced solubility and long-term stability over binary systems. Similarly, Saberi et al. (2023) demonstrated that Surfactant addition lowered surface tension and increased drug-medium interactions, thus increasing solubility and accelerating dissolution. This combination of ingredients allows TSDs to attain enhanced solubility relative to their binary counterparts, as the third component promotes improved molecular interaction and more efficient solubilization processes (Watanabe et al., 2002). Moreover, Saberi et al. (2023) also documented that the systems are more resistant to crystallization during storage and dissolution, overcoming the usual limitation of binary dispersions such that high content of polymers lowers miscibility and water sensitivity. These results indicate that TSDs are not incremental advancement in comparison to binary systems, but represent a more robust formulation design methodology. By combining multiple functionally complementary excipients, ternary systems can significantly enhance solubility, bioavailability, and stability, thereby providing tangible benefits for the therapeutic performance of poorly soluble drugs.

## 5 Development of ternary system based on amorphous solid dispersion

### 5.1 Drug-polymer polymer

An expanding body of literature has emphasized the strategic advantage of incorporating dual polymers within ternary amorphous solid dispersions (ASDs) to address the poor aqueous solubility of challenging drug molecules. Gala et al. (2020) conducted a longitudinal series of investigations on abiraterone-based ASDs processed via kinetisol, incorporating HPBCD alongside various secondary polymers. Among the series, the formulation containing HPMCAS126G, achieved the highest solubility at 82 μg/mL a 7.45-fold enhancement over the pure drug followed by systems with HPMCE50 (6.64-fold), HPMCE15 (6.18-fold), and HPMCAS912G (6.00-fold). Earlier iterations utilizing Eudragit L100–55 and PVPK90 demonstrated moderate outcomes, whereas those employing NACMC and PVPAP offered the least improvement (∼3.44-fold), underscoring the critical influence of polymer chemistry on solubility performance.

In parallel, Sarabu et al. (2022) reported consistent yet moderate 2.5-fold increases in atorvastatin solubility using PS630 and PEG400 via hot-melt extrusion. In contrast, Pöstges et al. (2022) observed a substantially greater 53.33-fold increase for celecoxib using a MA-EA–HPC matrix, demonstrating that polymer synergy can dramatically alter outcomes even within similar drug classes. Ishtiaq et al. (2024) evaluated curcumin-based ternary ASDs and found solubility enhancements ranging from 25.0-fold (Syloid 244) to 77.5-fold (Soluplus), highlighting pronounced formulation-dependent variability. Similarly, indometacin solubility improved by 10-fold (Wang et al. (2020); Eudragit–PVPK30) and 21.34-fold (Hanada et al. (2018a); HPMC–mesoporous silica), illustrating the benefit of combining matrix-forming polymers with porous carriers.

For itraconazole, Solanki et al. (2019) reported a marked disparity in solubility enhancement depending on the poloxamer used: Poloxamer 407 enabled a 41.49-fold increase, while Poloxamer 188 yielded only 20.75-fold. In comparison, Meng et al. (2017) documented a relatively modest 5.83-fold increase using a povidone-based system. Notably, Hörmann et al. (2018) achieved the most pronounced enhancement—666.67-fold—for nimodipine using a ternary HPMCE5–Eudragit system, outperforming all other reviewed formulations. Likewise, Müller et al. (2021) demonstrated a 61.67-fold improvement in regorafenib solubility through HPMCAS–povidone systems, regardless of polymer arrangement, confirming a strong and reproducible polymer synergy. These findings collectively affirm that polymeric pairings within ternary ASDs exert substantial influence over solubility performance and can be strategically optimized for enhanced biopharmaceutical properties.

### 5.2 Drug-polymer surfactant

Numerous studies have established that ternary ASDs integrating Surfactants can significantly enhance the solubility of poorly water-soluble drugs. Smith-Craven et al. (2024) utilized spray drying to combine Amphotericin B with HPMCAS912 and SDS, yielding a 168.86-fold increase in solubility. Yang et al. (2023), using solvent evaporation, formulated ritonavir with PVPVA and various Surfactants, achieving the highest solubility boost with span (810.81-fold), followed by SDS (567.57-fold) and tween (283.78-fold)—results that markedly surpassed the 22.22-fold increase reported by Wu et al. (2023), using PVPVA and span 20 via twin-screw extrusion. Siriwannakij et al. (2021) reported comparable, though lower, enhancements of 15.91-fold for ritonavir in systems combining PVPVA with either span 20 or poloxamer 407 using film casting, reflecting consistency with Wu's milder outcomes.

Moseson et al. (2023) found similar modest enhancements in bicalutamide solubility—6.63-fold with SDS and 6.51-fold with vitamin E TPGS—highlighting the reproducibility of Surfactant-based ternary systems using solvent evaporation. In contrast, Saboo et al. (2021) demonstrated more variable results for felodipine, ranging from 8.12-fold using DATPEGS via cryo-milling to 123.08-fold when TPGS was employed in a solvent-shift system, illustrating the critical influence of both method and Surfactant type. Rahman et al. (2020) reported relatively low increases for griseofulvin—2.12-fold (HPC–SDS) and 2.06-fold (Soluplus–SDS)—despite employing dual methods (spray drying and hot-melt extrusion), suggesting possible matrix limitations.

Solanki et al. (2019) achieved a 352.70-fold increase for itraconazole using HPMCAS–DATPEGS via hot-melt extrusion, outperforming Deshpande et al. (2018), whose best results reached 213.76-fold (HPMCAS-HF–TPGS), with other systems ranging from 199.60-fold (Soluplus–SLS) to only 17.64-fold (PVPVA–SLS), all prepared via spray drying. Meanwhile, Sarabu et al. (2022) documented consistent but modest 2.5-fold improvements for atorvastatin with PS-based polymers and Tween 80. Tung et al. (2021) reported a minimal 1.47-fold enhancement for L-tetrahydropalmatine using HPMCP and poloxamer 188. Altogether, these outcomes affirm the superior solubilizing potential of Surfactant-containing ternary ASDs, while also highlighting variability arising from Surfactant type, process method, and drug characteristics.

### 5.3 Drug-polymer excipient

The application of small molecule excipients in ternary ASDs has also proven effective in improving drug solubility across various formulation approaches. Czajkowski et al. (2023) employed freeze-drying to formulate fenofibrate with PVPVA and hydroxypropyl lecithin (HPL), achieving enhancements of 75.19-fold (from 1.33 μg/mL) and 21.19-fold (from 4.72 μg/mL), depending on initial solubility. Using cryogenic ball milling, Rosiak et al. (2024) formulated fisetin with Eudragit (EL100 or EPO) and hydroxypropyl-β-cyclodextrin, observing substantial solubility increases of 156.71-fold and 62.32-fold, respectively, further reinforcing the value of hydrophilic excipients in ternary systems.

Njoku et al. (2020) explored melt-based ternary ASDs comprising ritonavir, copovidone, and colloidal silicon dioxide under different pH conditions. Solubility enhancements varied significantly—1.68-fold at pH 1, but rising to 31-fold at pH 2 and 30-fold at pH 6.8—highlighting pH-dependent behavior. Compared to the more consistent and higher enhancements reported by Czajkowski and Rosiak, Njoku's findings suggest that the effectiveness of small molecule excipients such as silicon dioxide is more conditional and system-specific. Collectively, these results validate that incorporating judiciously selected excipients—particularly phospholipids and cyclodextrins—into ternary ASDs can yield significant solubility gains, although performance remains closely tied to both formulation strategy and the physicochemical context.

### 5.4 Drug-drug-polymer

Recent advances have expanded the concept of *co*-amorphous drug systems by incorporating a polymeric component, leading to the development of drug-drug-polymer ternary ASDs. The formation of this system has demonstrated considerable promise in enhancing the dissolution and apparent solubility of poorly water-soluble drugs. For instance, ternary systems composed of sulfamethoxazole (SMZ) and trimethoprim (TMP) were successfully prepared via the melt quenching method, employing Eudragit and PAA as polymeric carriers. Both systems exhibited substantial enhancement in solubility relative to their crystalline counterparts. Specifically, the SMZ:TMP:Eudragit formulation showed a 6.4-fold increase in SMZ dissolution and a 1.9-fold increase in TMP, whereas the SMZ:TMP:PAA system achieved 2.6-fold and 4.6-fold improvements, respectively (Li et al., 2022). Similarly, Riekes et al. (2016) developed a fixed-dose combination (FDC) of ezetimibe (EZE) and lovastatin (LOV) using the hyrdophilic polymer Soluplus® to improve solubility and dissolution. The formulation achieved rapid dissolution—92 % of EZE (18-fold) and 83 % of LOV (6-fold) within five minutes. These improvements are attributed to the formation of a molecularly homogeneous amorphous matrix that disrupts crystalline order and enhances wettability. Moreover, hydrogen-bonding and ionic interactions between the APIs and the polymers likely stabilize the amorphous phase, maintaining a transient supersaturation that promotes faster and more synchronized drug release.

### 5.5 Comparative analysis

**Fig. 2:**
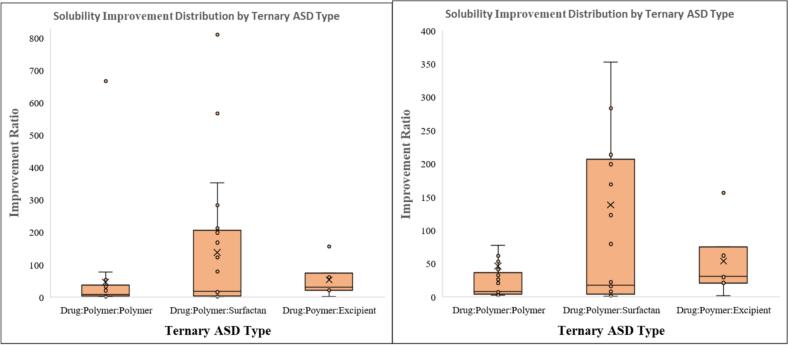
Solubility improvement distribution by amorphous solid dispersion system.

Fig. 2. Box and whisker plot representing the distribution of solubility improvement ratios across different ternary amorphous solid dispersion (ASD) systems: Drug:Polymer:Polymer, Drug:Polymer:Surfactant, Drug:Polymer:Excipient, and Drug:Drug:Polymer. The central line in each box indicates the median, the “×” marker represents the mean, and the upper and lower edges of each box denote the interquartile range (IQR). Whiskers extend to 1.5 × IQR, and individual points represent outliers. The Drug:Polymer:Surfactant system demonstrates the widest variability and the highest median and mean values, indicating a potentially superior capability in enhancing solubility, albeit with greater formulation variability. These findings suggest the composition of the ternary matrix significantly influences the magnitude of solubility enhancement.

Based on cumulative solubility enhancement data across ternary ASD systems, Smith-Craven et al. (2024) reported the highest improvement ratio of 168.86-fold for amphotericin B using a drug–polymer–Surfactant system formulated via spray drying with HPMCAS 912 and SDS. This outcome aligns with Yang et al. (2023), who reported even higher ratios for ritonavir formulated with PVPVA and Surfactants, achieving 810.81-fold with span, 567.57-fold with SDS, and 283.78-fold with tween through solvent evaporation. In contrast, Gala et al. (2020) achieved a 7.45-fold increase for abiraterone in a drug–polymer–polymer system using HPMCAS126G and HPBCD via kinetisol processing, which was the most potent among that subgroup, yet considerably lower than most Surfactant-based systems. Czajkowski et al. (2023), utilizing freeze drying, observed a 75.19-fold increase for fenofibrate using a drug–polymer–excipient system incorporating PVPVA and hydroxypropyl lecithin, representing a median enhancement between the Surfactant and polymer systems. Solanki et al. (2019) reached a 352.70-fold increase for itraconazole using HPMCAS and DATPEGS in a hot-melt extruded Surfactant-based system, which surpassed Müller et al. (2021), who reported a 61.67-fold improvement for regorafenib in a polymer–polymer matrix. In drug–polymer–excipient systems, Rosiak et al. (2024) achieved a 156.71-fold enhancement for fisetin using Eudragit and hydroxypropyl-β-cyclodextrin via cryogenic milling, placing the result close to the highest Surfactant formulations and exceeding most polymer-based counterparts. Pöstges et al. (2022) recorded a 53.33-fold improvement for celecoxib with a drug–polymer–polymer system, while Saboo et al. (2021) reported a 123.08-fold increase for felodipine in a Surfactant-based solvent shift system. Additionally, Drug–Drug–Polymer systems demonstrated remarkable synergistic enhancement, such as 18-fold and 6-fold increases for ezetimibe and lovastatin (Riekes et al., 2016), and 6.4-fold for sulfamethoxazole in combination with trimethoprim (Li et al., 2022). The range observed for Surfactant systems remained the broadest, with multiple formulations exceeding 200-fold, whereas polymer–polymer systems largely clustered below 100-fold. Excipient-based systems exhibited intermediate performance, with several formulations falling between 30- to 150-fold. Overall, solubility improvement distribution across studies was most pronounced in drug–polymer–Surfactant ASDs, followed by drug–polymer–excipient and drug–polymer–polymer systems, while drug–drug–polymer demonstrated relatively lower and narrower enhancement ranges.

### 5.6 The impact of ternary system amorphous solid dispersion on release profile

Gala et al. (2020) investigated a ternary ASD system composed of abiraterone, HPBCD, and HPMCAS 126G, revealing that the formulation achieved the highest dissolution and supersaturation levels, with approximately 60 μg/mL released after 90 min, outperforming both binary systems and other ternary variants. This result aligns with findings by Ishtiaq et al. (2024), where a ternary system of curcumin, HPMC E5, and Soluplus demonstrated superior dissolution at 91 % ± 3.89 %, markedly higher than pure curcumin, which only achieved 10 % ± 2.58 %. Similarly,

Hanada et al., 2018a, Hanada et al., 2018b reported immediate release performance with >85 % nimodipine dissolution within 30 min when using HPMCE5 and Eudragit in a ternary composition. In contrast, Müller et al. (2021) described a ternary regorafenib system (HPMCAS and Povidone) that exhibited slow release behavior (<10 μg/mL at 90 min) under gastric simulation, emphasizing the formulation's vulnerability to acidic environments despite statistically insignificant pharmacokinetic improvements. A distinctively prolonged supersaturation profile was documented by Hanada et al. (2018b) for indomethacin: HPMC: mesoporous silica, sustaining concentrations of 217.5 μg/mL over 24 h. Moving to polymer–Surfactant systems, Smith-Craven et al. (2024) demonstrated that ternary ASDs of amphotericin B with HPMCAS and SDS yielded up to a 90-fold increase in dissolution over the pure drug, whereas binary systems using a higher Surfactant concentration achieved only 40-fold enhancement. Sarabu et al. (2022) showed that atorvastatin ternary formulations with Tween 80 and PS530U/PS630 enabled >90 % release within 60 min, consistent with results by Wu et al. (2023), who reported similar performance for ritonavir systems utilizing PVPVA and Span 20. Yang et al. (2023) further established that ritonavir ternary ASDs with PVPVA and Span or SDS achieved full release (>100 %) within 45 min, in sharp contrast to Tween 80-based systems, which only reached >50 % by 90 min, suggesting excipient-dependent variability.

In studies of drug-drug-polymer system, sulfamethoxazole:trimethoprim:Eudragit and sulfamethoxazole:trimethoprim:PAA ternary ASDs prepared by the melt quenching method achieved rapid dissolution with approximately 80 % drug release within the first 10 min, confirming the effectiveness of both polymers in enhancing early-stage solubility (Li et al., 2022). Similarly, Trasi and Taylor (2015) reported that ritonavir:lopinavir ternary ASDs formulated with either PVP or HPMCAS achieved about 60 % drug release within 20 min, emphasizing the crucial role of polymer type in enhancing dissolution kinetics. Furthermore, Pacult et al. (2019) demonstrated that the flutamide:bicalutamide:PVP system achieved remarkable solubility improvements–2-fold for flutamide and 3-fold for bicalutamide–although their dissolution rates (25 % and 5 % after 60 min, respectively) differed due to intrinsic physicochemical disparities between drugs. These findings confirm that incorporating a polymeric component into drug–drug systems consistently improve solubility and dissolution performance, even though the extent of enhancement may vary among co-formulated drugs.

In the case of polymer–excipient systems Czajkowski et al. (2023) formulated fenofibrate with PVPVA and HPL, observing high initial supersaturation (∼100 μg/mL) in both FaSSIF and FeSSIF media, yet dissolution concentrations declined by 60 min due to recrystallization. Complementarily, Njoku et al. (2020) reported that ritonavir ternary systems containing colloidal silicon dioxide showed pH-dependent dissolution, with >60 % released in 60 min at pH 1.0 and > 50 % at pH 2.0, while neutral pH (6.8) limited release to ∼30 %, reflecting modest to moderate enhancement depending on gastrointestinal environment. These comparative findings collectively illustrate that while ternary ASD systems consistently improve drug release profiles across various formulations, the extent and sustainability of supersaturation are strongly influenced by the type of third component incorporated.

### 5.7 The impact of ternary system amorphous solid dispersion on drug bioactivity and pharmacological properties

The evaluation of ternary system amorphous solid dispersions (ASDs) on pharmacological performance, including bioavailability, permeability, and biological activity, reveals a consistent trend of improved drug bioactivity across different ternary compositions. For the Drug:Polymer:Polymer system, Gala et al. (2020) demonstrated that the ternary ASD of abiraterone with HPBCD and HPMCAS126G significantly enhanced its pharmacokinetic profile, resulting in a 13.8-fold increase in bioavailability compared to the commercial abiraterone acetate tablet, outperforming the binary system which achieved a 12.4-fold increase. Similarly, Ishtiaq et al. (2024) showed that curcumin formulated with HPMC E5 and Soluplus not only improved dissolution but also significantly enhanced anti-inflammatory, anti-oxidant, and antibacterial activities in the F3 formulation compared to the pure compound. Although Wang et al. (2020) did not report in vivo data, their in vitro analysis indicated a strong concentration-dependent inhibition of indomethacin precipitation, suggesting potential in vivo advantages. For the Drug:Polymer:Surfactant system, Tung et al. (2021) reported that the inclusion of poloxamer 188 in the ternary ASD of L-tetrahydropalmatine led to notable improvement in pharmacokinetics, where Cmax and AUC increased markedly compared to the pure drug, albeit with slightly lower performance than the binary counterpart, indicating the role of Surfactant in modulating bioavailability. Within the Drug:Polymer:Excipient group, Czajkowski et al. (2023) found that ternary ASDs of fenofibrate incorporating HPL significantly enhanced in vitro permeation, achieving 2.25 μg of final mass permeated, comparable or superior to commercial formulations. Furthermore, Rosiak et al. (2024) highlighted that fisetin ternary ASDs with Eudragit EL100 or EPO and HPβCD exhibited a substantially enhanced antioxidant and neuroprotective effect, with the 30 % loaded ASI_30_EPO variant being the most potent, indicating a synergistic effect of both excipients on bioactivity. Additionally, in the Drug:Drug:Polymer system, Naama et al. (2025) ternary ASDs of cefdinir-curcumin-PVP exhibited approximately a 1.833-fold enhancement in antibacterial efficacy compared to the pure drug, demonstrating that this system also potentiates synergistic pharmacological activity. These results collectively underscore that ternary ASDs—regardless of their specific configuration—can substantially improve pharmacological properties, with variations in effectiveness depending on the API characteristics and the nature of incorporated excipients.

## 6 Discussion

The integration of ternary systems in the design of amorphous solid dispersions (ASDs) has emerged as a transformative approach for overcoming the biopharmaceutical limitations of poorly water-soluble drugs. This review confirms that all four ternary systems—Drug–Polymer–Polymer, Drug–Polymer–Surfactant, Drug–Polymer–Small Molecule Excipient, and Drug–Drug–Polymer—exhibit notable improvements in solubility, dissolution behavior, and pharmacological properties compared to their binary counterparts. However, the magnitude of enhancement and the mechanistic drivers behind each system differ substantially, indicating that formulation success is highly context-dependent and relies on rational excipient selection and design.

**Fig. 3:**
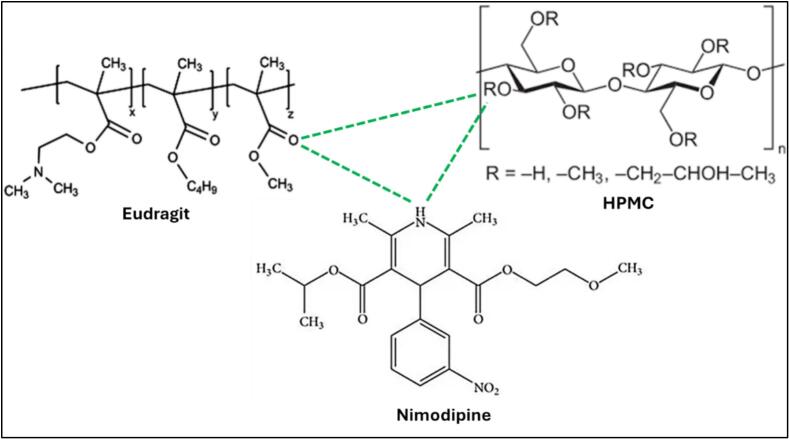
Molecular Interaction of Drug-Polymer-Polymer Amorphous Solid Dispersion System.

The Drug–Polymer–Polymer systems, although generally less potent in solubility improvement compared to Surfactant-containing systems, exhibit significant enhancements through synergistic polymer–polymer interactions that stabilize the amorphous state and inhibit recrystallization. Hörmann et al. (2018) formulated an amorphous solid dispersion (ASD) of nimodipine employing a dual-polymer matrix composed of Eudragit E and HPMC E5 via hot-melt extrusion (HME) followed by strand pelletization. The study demonstrated that nimodipine molecules were predominantly solubilized within the Eudragit E polymeric fraction. A distinct shift of the carbonyl (C=O) stretching band of nimodipine upon mixing with Eudragit E indicated the formation of hydrogen bonding between the carbonyl groups of nimodipine and the tertiary amine group of Eudragit E. In the ternary system, this characteristic band remained shifted but exhibited slight broadening, suggesting that hydrogen bonding interactions persisted, although they were partially modulated by the hydroxyl groups of HPMC E5. While HPMC E5 did not engage in strong direct interactions with nimodipine, it influenced the polymeric environment through physical stabilization. HPMC E5 contributed to the formation of a viscous matrix that retarded nimodipine diffusion and enhanced the amorphous stability of the system. Collectively, the dual-polymer system provided complementary functional roles: Eudragit E served to maintain nimodipine in an amorphously dispersed state, whereas HPMC E5 acted as a thermodynamic stabilizer preventing phase separation and recrystallization (Fig. 3). For instance, combinations like HPMCAS with HPBCD or povidone can effectively elevate the glass transition temperature (Tg) of the matrix, thereby reducing molecular mobility and impeding nucleation events. The hydrophilic-hydrophobic balance between polymers plays a crucial role, where the hydrophobic segments enhance drug-polymer miscibility, and the hydrophilic regions promote wettability and dispersion. Additionally, steric hindrance provided by long polymer chains can suppress aggregation and precipitation of the drug upon dissolution. The performance variation among polymer combinations—e.g., Eudragit vs. HPMC vs. Soluplus—can be attributed to their distinct hydrogen-bonding capacities, molecular weights, and functional group architectures, all of which govern their ability to form strong intermolecular interactions with the API. Nevertheless, the relatively lower solubility improvement in some systems arises from the lack of interfacial activity, which limits wetting and fails to reduce surface energy sufficiently for rapid drug dispersion.

**Fig. 4:**
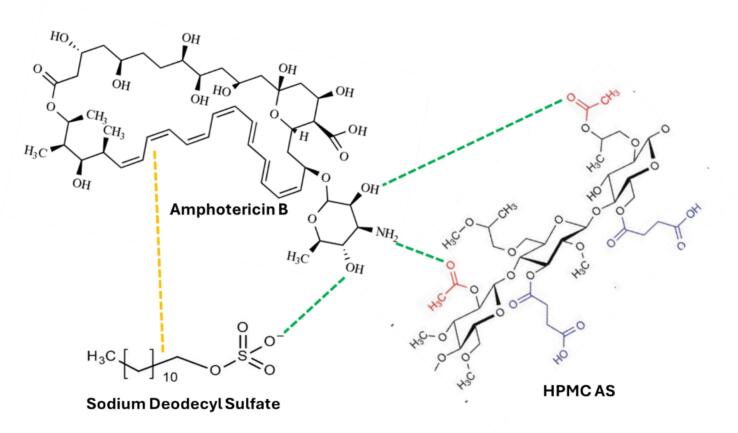
Molecular Interaction of Drug-Polymer-Surfactan Amorphous Solid Dispersion System.

**Fig. 5:**
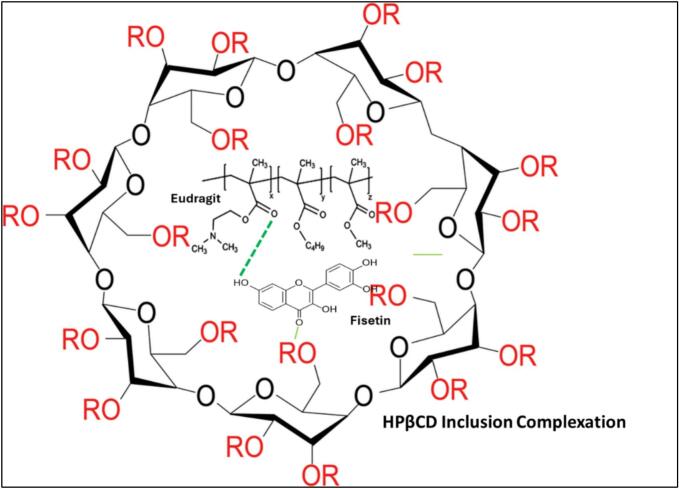
Molecular Interaction of Drug-Polymer-Excipient Amorphous Solid Dispersion System.

In contrast, the Drug–Polymer–Surfactant systems consistently outperformed all other formulations, with several reports exceeding 100- to 800-fold increases in solubility. This remarkable enhancement is primarily driven by interfacial and micellization phenomena. Surfactants such as SDS, Span, and TPGS dramatically reduce surface tension, improving drug wetting and dispersion in aqueous environments. Moreover, these Surfactants can form micelles or mixed micellar structures with polymers, effectively encapsulating hydrophobic drug molecules and increasing their apparent solubility. Smith-Craven et al. (2024) reported the development of a ternary formulation of amphotericin B comprising HPMCAS and sodium dodecyl sulfate. In crystalline form, amphotericin B exhibits strong intermolecular interactions (hydrogen bonding and van der Waals) that promote molecular aggregation, thereby limiting contact with the dissolution medium and reducing the dissolution rate. In the ternary system, multiple intermolecular interactions are possible, including hydrogen bonding (e.g., –OH or –NH groups of the drug with the carbonyl or ether groups of the polymer), dipole–dipole, ion–dipole (particularly in the presence of charged surfactants), and hydrophobic interactions. The alkyl chain of the surfactant interacts with the lipophilic polyene chain of amphotericin B, while the negatively charged sulfate group of surfactant associates with polar or charged regions of the drug. These interactions facilitate the formation of a localized “microenvironment” that enhances drug dissolution—potentially through the generation of micelles structures surrounding the drug molecules. Meanwhile, the polymer HPMCAS functions as a structural “matrix” that immobilizes the dispersed drug, thereby preventing molecular mobility and recrystallization. The collective interactions among the drug, polymer, and surfactant result in the formation of an unified matrix, as evidenced by a single peak (∼895 nm) observed in dynamic light scattering analysis, indicating that all components coexist within a single aggregated phase rather than as separate entities (Fig. 4). (See Fig. 5.)

The variability observed between different Surfactants is closely related to their critical micelle concentration (CMC), hydrophilic–lipophilic balance (HLB), and ionic nature. For example, SDS, an anionic Surfactant with high solubilization capacity, promotes significant disruption of the drug's crystal lattice, whereas nonionic Surfactants like Tween or Span may exert milder effects depending on their HLB and steric stabilization capability. Furthermore, the Surfactant's capacity to enhance drug permeability through modulation of membrane fluidity or inhibition of efflux transporters (e.g., P-gp) may also explain the higher bioactivity observed in Surfactant-based ternary ASDs. Despite this, not all Surfactant systems sustain supersaturation effectively—some promote rapid initial release but allow recrystallization due to insufficient polymeric stabilization, underlining the importance of polymer–Surfactant compatibility.

The Drug–Polymer–Small Molecule Excipient systems demonstrated intermediate performance, with several systems achieving >150-fold solubility improvement. This enhancement is attributed to molecular-level interactions and physicochemical modifications imparted by functional excipients such as cyclodextrins, phospholipids, or silicon dioxide. Rosiak et al. (2024) developed an amorphous solid dispersion (ASD) system of fisetin with Eudragit polymer using a solvent-free mechanochemical approach via ball milling. Subsequently, a ternary system was formulated by incorporating 2-hydroxypropyl-β-cyclodextrin (HPβCD). The FTIR analysis of pure fisetin revealed characteristic absorption bands within the range of approximately 400–1800 cm^−1^ (attributable to skeletal vibrations of the flavonoid structure) and 3200–3600 cm^−1^ (corresponding to –OH stretching vibrations). Eudragit polymer exhibited typical ester/carbonyl (C=O) and C–O–C or C—O stretching bands associated with carboxylate and ester functional groups. In this system, molecular interactions were observed between fisetin and the Eudragit matrix. The –OH groups of fisetin (notably those located on rings A or B of the flavonoid structure) were found to participate in hydrogen bonding with the ester carbonyl groups of Eudragit®. Molecular modeling further confirmed the potential formation of a hydrogen bond between the –OH group of fisetin and the oxygen atom of the carbonyl (O=C) moiety of Eudragit®. Moreover, the fisetin–Eudragit complex was partially accommodated within the cavity of HPβCD (inclusion complexation) or interacted at its outer surface. HPβCD also exhibited the ability to form hydrogen bonds or other non-covalent interactions with either the polymer or the drug through its C—O and C—H groups. The observed shift of the HPβCD absorption band from 1006 cm^−1^ to 1028 cm^−1^ confirmed the involvement of its C–O/C–H groups in the molecular interactions within the ternary system. Overall, the Eudragit polymer provided a stabilizing matrix that maintained the drug in an amorphous state, whereas HPβCD enhanced solubility through inclusion complexation and additional molecular-level interactions.

Cyclodextrins (e.g., HPβCD) enhance solubility via inclusion complexation, where the hydrophobic cavity encapsulates the drug, shielding it from the aqueous environment and enhancing dissolution. Phospholipids, such as hydroxypropyl lecithin, improve drug dispersion and membrane permeability by mimicking biological bilayers, while colloidal silicon dioxide facilitates adsorptive stabilization and particle size reduction. These small excipients not only promote wettability but also help maintain supersaturation by forming micro-domains around drug particles. However, the observed variability—such as pH-dependent performance in silicon dioxide-based systems—highlights the conditional efficacy of such excipients, which are often sensitive to environmental factors and formulation architecture.

The Drug–Drug–Polymer systems demonstrated intermediate yet notable performance, with several formulations achieving up to 18-fold increases in solubility, accompanied by substantial improvements in dissolution and pharmacological efficacy. These enhancements are primarily driven by strong intermolecular interactions between the drugs and the polymer, which stabilize the amorphous matrix and maintain local supersaturation. Li et al. (2022) reported that SMZ:TMP:Eudragit and SMZ:TMP:PAA ternary ASDs exhibited rapid dissolution (>80 % within 10 min), reflecting efficient early-stage solubilization mediated by polymer–drug interactions. Likewise, Trasi and Taylor (2015) and Pacult et al. (2019) observed enhanced dissolution in RTV:LPV and FL:BIC systems, indicating that polymer incorporation not only inhibits nucleation and crystal growth through surface adsorption but also harmonizes the release profiles of both drugs. This synchronized release accelerates dissolution kinetics and promotes concurrent drug availability, thereby amplifying pharmacodynamic synergy. However, despite these synergistic advantages, certain ternary systems may exhibit reduced amorphous solubility due to decreased chemical potential, indicating that the enhancement in pharmacological performance may occur at the expense of solubility capacity.

Beyond classical supersaturation dynamics, recent studies have emphasized the critical role of liquid–liquid phase separation (LLPS) in governing the dissolution and stability behavior of ASDs. LLPS refers to the spontaneous demixing of a supersaturated drug solution into a drug-rich and a drug-poor (continuous) phase before precipitation occurs. In binary ASDs, LLPS typically arises when molecular interactions between the drug and polymer are insufficient to maintain a homogeneous supersaturated solution, leading to nanoscale droplet formation. These drug-rich droplets act as kinetic reservoirs that transiently sustain apparent solubility; however, they often coalesce or crystallize over time due to inadequate polymeric stabilization. In contrast, ternary systems—particularly those incorporating surfactants or secondary polymers—demonstrate enhanced control over LLPS, where the additional component localizes at the interface of drug-rich domains, reducing interfacial tension and inhibiting droplet coalescence. For instance, surfactants lower the energy barrier for dispersion of these nano-droplets, while hydrophilic *co*-polymers form hydrogen-bond networks that immobilize the drug within the continuous phase. This synergistic modulation of LLPS effectively delays or prevents drug crystallization, thereby extending the duration of supersaturation and improving dissolution efficiency. Consequently, the suppression or stabilization of LLPS represents a defining mechanistic advantage of ternary ASDs over their binary counterparts, translating molecular-level miscibility into measurable biopharmaceutical benefits such as prolonged supersaturation, enhanced dissolution rate, and improved absorption profiles.

Finally, the evaluation of pharmacological outcomes confirmed that improvements in solubility and release directly translated to enhanced bioactivity. Drug–polymer–polymer systems improved bioavailability through sustained release and stabilization effects, as observed with abiraterone and curcumin. Surfactant-containing systems, such as those for L-tetrahydropalmatine and ritonavir, demonstrated enhanced Cmax and AUC, facilitated by improved solubilization and possibly membrane interaction effects. Excipient-based systems, particularly those using HPβCD and phospholipids, not only improved in vitro permeability but also augmented pharmacodynamic properties such as antioxidant and neuroprotective activity, likely due to more efficient cellular uptake or protection from degradation. In addition, Drug–Drug–Polymer systems further enhanced therapeutic outcomes by enabling simultaneous delivery of two synergistic actives, translating physicochemical improvements into amplified pharmacodynamic responses.

In summary, ternary ASDs represent a multifaceted strategy to enhance the solubility, dissolution, and pharmacological performance of poorly water-soluble drugs. The superior efficacy of Surfactant-based systems lies in their capacity to simultaneously enhance solubilization, improve dispersion, and modulate biological membranes. Polymer–polymer systems offer stable supersaturation and crystallization inhibition but are limited by their lack of interfacial activity. Excipient-based systems exploit molecular-level interactions to improve local solubility and bioactivity, although their performance is more environment-dependent. Drug–Drug–Polymer systems enable synergistic pharmacological effects by co-formulating drugs with complementary mechanisms within a stabilized amorphous matrix, though this benefit can be compromised by reduced amorphous solubility or formulation instability arising from intermolecular competition between the incorporated drugs. Collectively, these insights emphasize the necessity of rational excipient selection, guided by both physicochemical understanding and mechanistic rationale, to optimize ternary ASD formulations for maximum biopharmaceutical benefit.

### 6.1 Mechanism perspective

**Fig. 6:**
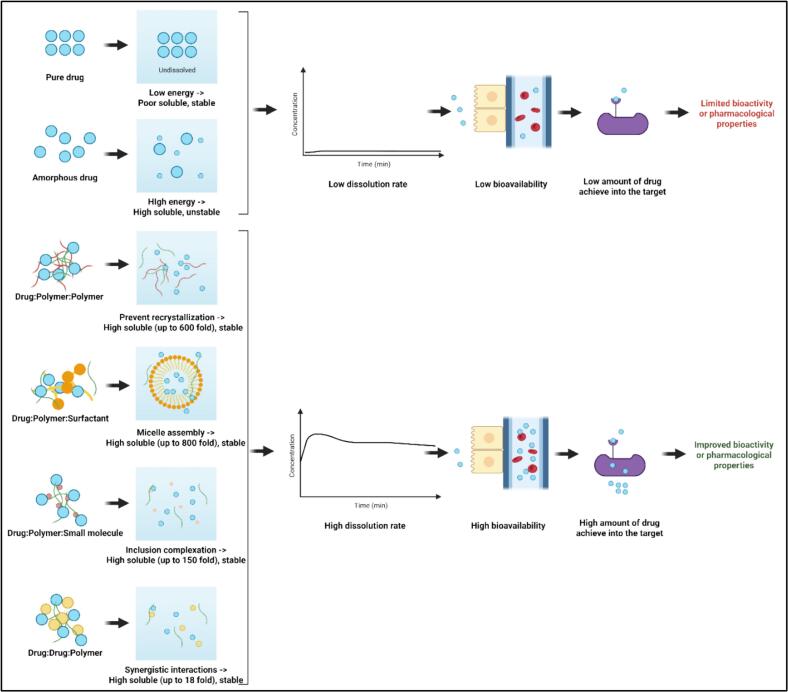
Mechanism insight.

The remarkable enhancement in drug solubility, dissolution, stability, and pharmacological performance observed in ternary amorphous solid dispersion (ASD) systems can be mechanistically explained through a multilayered molecular interaction network established between the drug, primary polymer, and a secondary excipient whether it be a surfactant, an additional polymer, or a small molecule (Fig. 6). At the core, the amorphous state of the drug, inherently more soluble due to its higher Gibbs free energy compared to the crystalline form, is thermodynamically stabilized by strong intermolecular interactions such as hydrogen bonding, dipole-dipole forces, and van der Waals interactions with polymer matrices. This transformation effectively enhances the drug's apparent solubility and creates a high-energy, supersaturated state.

Beyond compositional variations, the preparation technique exerts a profound influence on the molecular interactions, amorphous stability, and dissolution performance of ternary amorphous solid dispersions (ASDs). Comparative findings from recent studies indicate that each preparation method such as spray drying, hot-melt extrusion, solvent evaporation, melt quenching, and freeze drying imposes distinct thermodynamic and kinetic conditions that govern drug–polymer miscibility, molecular dispersion, and crystallization inhibition. Spray-dried ternary systems, for instance, typically exhibit superior solubility enhancement and rapid dissolution owing to the formation of smaller, more homogeneous particles and stronger surface wetting, particularly when optimized solvent systems are used (Zhang et al., 2025). In contrast, hot-melt extrusion promotes intimate molecular mixing through elevated temperature and shear stress, facilitating stronger hydrogen bonding and enhanced amorphous stability, as observed in nimodipine and itraconazole ternary ASDs (Sharma et al., 2018). Meanwhile, solvent evaporation and freeze-drying techniques allow greater control over solvent–polymer interactions, yielding high supersaturation but often at the expense of reduced long-term stability due to residual solvent or moisture content (Czajkowski et al., 2023). Collectively, these observations emphasize that the preparation method not only determines the physical characteristics of the ASD but also modulates the extent and strength of drug–polymer–excipient interactions at the molecular level. Thus, achieving optimal solubility and stability in ternary systems requires careful alignment between formulation composition and preparation methodology to maximize synergistic effects and maintain sustained supersaturation.

The incorporation of a surfactant into the ternary matrix amplifies this enhancement by introducing amphiphilic domains that reduce interfacial tension and dramatically increase drug wettability. This action facilitates the rapid hydration of the matrix and promotes the formation of micelles or mixed micellar assemblies that act as dynamic nanocarriers encapsulating drug molecules and shielding them from precipitation. These micellar structures not only serve as solubilizing agents but also function as physical barriers that hinder molecular reordering, thereby kinetically suppressing nucleation and recrystallization. This dual mechanism improved wetting and micellar stabilization was profoundly evident in the work of Yang et al. (2023) and Solanki et al. (2019), where Drug:Polymer:Surfactant systems such as Ritonavir:PVPVA:Span and Itraconazole:HPMCAS:DATPEGS achieved extraordinary solubility enhancements of up to 810-fold and 352-fold, respectively values far exceeding those observed in systems lacking surfactant components.

In contrast, systems incorporating a second polymer contribute primarily through matrix densification and hydrogen-bonding reinforcement, leading to lower molecular mobility and increased thermodynamic stability. These attributes extend supersaturation duration and prevent recrystallization by restricting drug diffusion, as demonstrated in the study by Ishtiaq et al. (2024) using a curcumin:HPMC:Solupus ternary matrix, which provided both stability and significant antibacterial and antioxidant bioactivity. Small molecule excipients, such as cyclodextrins and colloidal silica, offer an orthogonal stabilization mechanism through inclusion complexation or surface adsorption. These interactions spatially isolate the drug and reduce its exposure to water or enzymatic environments, as seen in Rosiak et al. (2024) and Czajkowski et al. (2023), where ternary ASDs significantly improved fisetin's antioxidant activity and fenofibrate's permeation, respectively. Similarly, Drug–Drug–Polymer systems, as reported by Li et al. (2022) and Naama et al. (2025), exhibited synergistic enhancement in both dissolution and pharmacological activity, where two complementary drugs within a polymeric matrix promoted synchronized release, improved molecular miscibility, and amplified bioactivity beyond that of individual components.

Nonetheless, among all ternary architectures, Drug:Polymer:Surfactant systems consistently demonstrate the most potent solubility enhancement. This superiority stems from their ability to simultaneously address multiple biopharmaceutical barriers rapid matrix hydration, micelle-mediated solubilization, inhibition of recrystallization, and often, permeability enhancement via modulation of membrane transporters or paracellular pathways. This is strongly reflected in the solubility distribution data, where Drug:Polymer:Surfactant formulations dominate the upper quartile of improvement values, frequently surpassing 200–800 fold increases, highlighting their unparalleled capacity in enhancing the dissolution profile of poorly soluble compounds. These synergistic mechanisms cumulatively generate a steep and sustained concentration gradient across biological membranes, facilitating superior drug absorption.

Ultimately, these cascading effects converge into meaningful pharmacokinetic advantages elevated Cmax, enhanced AUC, and reduced Tmax which directly correlate with heightened therapeutic exposure. Hence, ternary ASDs, particularly those employing surfactants, represent not only a molecularly rational strategy for solubility enhancement but a transformative platform for overcoming complex delivery challenges in oral drug administration.

## 7 Conclusions and future perspective

This comprehensive review has successfully addressed its primary objective of elucidating the strategic function and mechanistic foundations of ternary amorphous solid dispersion (ASD) systems in improving the solubility, dissolution, stability, and pharmacological efficacy of poorly water-soluble drugs. Systematics comparison of drug–polymer–polymer, drug–polymer–Surfactant, drug–polymer–excipient, and drug–drug–polymer systems reveal that the addition of a third component, especially Surfactants or functional excipients, enhances performance beyond that of binary systems via multifaceted molecular mechanisms. These encompass improved wettability, molecular dispersion, micellization, hydrogen bonding, steric stabilization, and recrystallization inhibition, all of which synergistically maintain supersaturation and promote enhanced absorption. The rational design of ternary ASD systems, based on molecular compatibility and formulation techniques, has significant potential to overcome biopharmaceutical limitations and optimize therapeutic efficacy. As a result, ternary ASD systems represent a robust and pharmacologically significant scientific advancement in modern drug delivery technology.

## CRediT authorship contribution statement

**Arif Budiman:** Writing – review & editing, Writing – original draft, Validation, Supervision, Methodology, Funding acquisition, Conceptualization. **Lisa Efriani Puluhulawa:** Writing – original draft, Software, Resources, Methodology, Investigation, Formal analysis. **Faradila Ratu Cindana Mo’o:** Writing – original draft, Visualization, Software, Resources, Investigation, Formal analysis. **Nurain Thomas:** Writing – review & editing, Visualization, Validation, Resources, Data curation. **Melvern Theodorik S. Biu:** Writing – original draft, Visualization, Software, Resources, Investigation, Formal analysis, Data curation. **Febrina Amelia Saputri:** Writing – review & editing, Validation, Supervision, Resources, Formal analysis. **Siti Farah Rahmawati:** Writing – review & editing, Validation, Supervision, Resources, Data curation. **Diah Lia Aulifa:** Writing – review & editing, Supervision, Project administration, Investigation, Conceptualization. **Salma Amaliah:** Writing – original draft, Visualization, Validation, Resources, Project administration, Investigation, Formal analysis, Data curation. **Agus Rusdin:** Writing – original draft, Visualization, Software, Resources, Project administration, Investigation, Data curation.

## Funding

This research was funded by Universitas Padjadjaran (Riset Kolaborasi Indonesia (RKI)) to A.B. (No. 818/UN6.3.1/PT.00/2025, 14 April 2025).

## Declaration of competing interest

The authors declare that they have no known competing financial interests or personal relationships that could have appeared to influence the work reported in this paper.

## Data Availability

No data was used for the research described in the article.
